# Fungal Alternative Splicing is Associated with Multicellular Complexity and Virulence: A Genome-Wide Multi-Species Study

**DOI:** 10.1093/dnares/dst038

**Published:** 2013-10-11

**Authors:** Konrad Grützmann, Karol Szafranski, Martin Pohl, Kerstin Voigt, Andreas Petzold, Stefan Schuster

**Affiliations:** 1 Department of Bioinformatics, Friedrich Schiller University Jena, Ernst-Abbe-Platz 2, Jena D-07743, Germany; 2 Genome Analysis, Leibniz Institute for Age Research – Fritz Lipmann Institute, Jena, Germany; 3 Jena Microbial Resource Collection, Leibnitz Institute for Natural Product Research and Infection Biology - Hans-Knöll-Institute, Jena, Germany; 4 Leibniz Institute for Natural Product Research and Infection Biology – Hans-Knöll-Institute, Jena, Germany

**Keywords:** alternative splicing, fungal genomes, transcriptome analysis, multi-cellular complexity, retained intron

## Abstract

Alternative splicing (AS) is a cellular process that increases a cell's coding capacity from a limited set of genes. Although AS is common in higher plants and animals, its prevalence in other eukaryotes is mostly unknown. In fungi the involvement of AS in gene expression and its effect on multi-cellularity and virulence is of great medical and economic interest. We present a genome-wide comparative study of AS in 23 informative fungi of different taxa, based on alignments of public transcript sequences. Random sampling of expressed sequence tags allows for robust and comparable estimations of AS rates. We find that a greater fraction of fungal genes than previously expected is associated with AS. We estimate that on average, 6.4% of the annotated genes are affected by AS, with *Cryptococcus neoformans* showing an extraordinary rate of 18%. The investigated Basidiomycota show higher average AS rates (8.6%) than the Ascomycota (6.0%), although not significant. We find that multi-cellular complexity and younger evolutionary age associate with higher AS rates. Furthermore, AS affects genes involved in pathogenic lifestyle, particularly in functions of stress response and dimorphic switching. Together, our analysis strongly supports the view that AS is a rather common phenomenon in fungi and associates with higher multi-cellular complexity.

## 1. Introduction

Via alternative splicing (AS) different mRNA isoforms are produced from one single gene. This diversification is one explanation for the discrepancy between the relatively low gene numbers of higher eukaryotes on the one hand and their cellular complexity on the other hand. AS affects binding properties, intracellular localization, enzymatic activity and many more properties of proteins.^1^ Examples of regulated pathways are sex determination in *Drosophila melanogaster*,^2^ neuronal differentiation in rat^3^ and auto-regulation of LAMMER kinases, which take part in splicing factor activation.^4^ Not only the mere presence of an isoform, but also the exact splice isoform ratio can influence the phenotype of cells and can be regulated in a tissue-dependent manner.^5^

Expressed sequence tags (ESTs) have widely been used to detect AS and quantify the transcript diversity arising from AS. For example, AS estimates for animals range from 53% of the multi-exon genes in human, 53% in mouse, 24% in rat, 22% in chicken, 19% in fruit fly to 6% in roundworm.^6^ Interestingly, despite their relatedness, the estimates for mouse and rat differ remarkably. A possible reason for this are too few transcript data that limit the detection of AS events. Therefore, an approach was suggested that corrects for the amounts of transcripts and yields similar AS rates of ∼31% for mouse and rat.^7^ Finally, from deep transcriptome sequencing an AS rate of >90% was estimated for humans.^8^ These findings support the view that sensitive methods will ultimately detect splicing variants for every multi-exon gene.^9^

The basic AS types are the following: in exon skipping (SE, cassette exon), the exon can be spliced out of the transcript together with its flanking introns.^10^ Alternative 5′ splice site (A5′SS, alternative donor) selection^11^ and alternative 3′ splice site (A3′SS, alternative acceptor) selection^12^ result in longer exons and corresponding isoforms.^13^ Intron retention describes a mechanism where an intron can remain in the mature mRNA.^14^ Previous studies showed that eukaryote species do not have equal distributions of these AS types. Cassette exons predominantly occur in animals, whereas intron retention is more frequent in other taxa.^15^

Fungi, especially *Saccharomyces cerevisiae*, have been used extensively as a reduced and easily manageable model system in biological research. There are many fungi that cause human and plant diseases (Supplementary Table S1), which provoke worldwide costs of several billion dollars a year. Other fungi are used for industrial fermentation and production of food and feed additives or are crucial in the degradation of xenobiotics and in the conversion of cellulose into biofuels (Supplementary Table S1). Fungi have compact genomes (the majority 10–90 Mbp) and genes with small introns. They also show extended consensus sequences for the 5′SS and the branchpoint region.^16^ These features facilitate a structural interpretation of intron sequences, and they suggest low-complex AS patterns, both of which make fungi attractive models for mechanistic studies of (alternative) splicing.

A few studies estimated fungal AS rates on a genome-wide scale in comparative manner. Varying but relatively low AS frequencies were discovered in fungi and microsporidia (0–5% of genes in *S. cerevisiae*, *Schizosaccharo-myces pombe*, *Encephalitozoon cuniculi* and *Cryptococcus neoformans*).^17^ A correspondence between intron numbers per gene and AS numbers was found for the studied species. Also, AS seems to affect genes of different functions. Genes associated with regulation have higher AS levels. Evolutionarily old genes were found to be affected more often.^17^ In another comparative study of 14 fungi among other eukaryotes, also varying amounts of AS were observed. Yeasts showed nearly no events, and around 1000 AS instances were found for *C. neoformans* and *Coccidioides posadasii*, each.^15^ Studies of single fungal species show results from only a few AS events in *Magnaporthe grisea*^18^ to rates of 8.6% in *Aspergillus oryzae*^19^ and 4.2% in *C. neoformans*.^20^ Remarkably, Ho *et al*.^21^ estimate an AS rate for *Ustilago maydis* of 26% in a subset of multi-exon genes that have support by at least two ESTs.

So far, AS research was mainly focused on animals and plants. With this study, we give a comprehensive report on fungi as the third eukaryote crown group. The comparability of the previous results on fungal AS is hampered due to the application of different biochemical and computational strategies. Thus, we undertook a systematic genome-wide comparative analysis of AS in 23 informative fungal species. The basis of our analysis are alignments of transcript sequences to genome sequences, and an AS rate estimation similar to that of Kim *et al*.^7^

## 2. Materials and methods

### 2.1. Data sources and preparation

We downloaded chromosomal sequences, reference transcripts and gene annotations of 25 species (26 different strains) from NCBI's GenBank, RefSeq and EntrezGene databases, respectively.^22^ These data were complemented with most up-to-date sequences and annotations of *Pichia stipitis* (Joint Genome Institute^23^) and *S. cerevisiae* (*Saccharomyces* genome database^24^). Genome sequences and annotations of further three species (five strains) were from the Broad Institute (*Fusarium oxysporum*, *Paracoccidioides brasiliensis* Pb01, Pb03 and Pb18, *Rhizopus oryzae*^25^) and for further three species from the Joint Genome Institute (*Phanerochaete chrysosporium*, *Trichoderma reesei*, *Mycosphaerella graminicola*^23^). ESTs for all species were downloaded from NCBI's dbEST database, except for *Arthroderma benhamiae*, where Roche 454 data are from NCBI's SRA.^22^ Four species were excluded from the analysis because there were <200 ESTs. This yielded 27 species (30 strains) with sufficient data (Table 1). We masked low-complexity repeats from the genome sequences using the program RepeatMasker (Smit *et al*., unpublished). We removed sequence contamination, low-quality and low-complexity sequences from the ESTs using SeqClean (unpublished, ‘The Gene Index Project’ of Harvard University). Roche 454 reads were additionally cleaned for adapter stretches using in-house software.

**Table 1.** Annotation, EST mapping and AS data of the studied species

Taxon^a^	Species	Lifestyle^b^	Annotated genes	Annotated introns	Number of available reads	% Filtered and mapped reads	% Genes covered with ≥2 reads	RIs	Skipped exons	Alternative 5′ intron ends	Alternative 3′ intron ends	% Genes w. any type of AS
A	*Ajellomyces capsulatus*	HP	9314	16 275	26 389	55	11	51	2	5	15	6.5
A	*Arthroderma benhamiae*	HP	7984	10 332	1 040 774	86	86	1381	68	292	445	8.2
A	*Chaetomium globosum*	HP	11 048	17 396	1557	34	1	1	0	0	0	
A	*Coccidioides immitis*	HP	10 440	17 815	62 729	93	49	664	18	152	225	13.4
A	*Paracoccidioides brasiliensis* Pb01	HP	9132	28 179	41 463	75	33	235	23	67	110	15.4
A	*Paracoccidioides brasiliensis* Pb03	HP	7875	19 575	41 463	71	35	134	16	31	52	10
A	*Paracoccidioides brasiliensis* Pb18	HP	8741	24 498	41 463	71	32	134	15	31	52	10.5
A	*Aspergillus nidulans*	NP	9541	16 797	16 848	89	15	81	1	11	14	7.3
A	*Aspergillus niger*	NP	10 597	17 668	46 938	91	28	323	7	37	43	9.5
A	*Aspergillus oryzae*	NP	12 823	20 916	9051	94	9	70	2	5	12	
A	*Neurospora crassa*	NP	9841	14 323	277 147	83	52	511	57	128	164	8.8
A	*Pichia stipitis*	NP	5807	2580	19 621	95	21	0	0	0	0	0
A	*Podospora anserina*	NP	10 257	11 261	51 862	92	30	194	5	43	83	4.8
A	*Saccharomyces cerevisiae*	NP	5781	332	34 915	97	41	2	0	2	7	0.18
A	*Schizosaccharomyces pombe*	NP	5073	3878	8123	78	10	3	0	0	0	0.6
A	*Trichoderma reesei*	NP	9143	18 802	44 964	76	40	66	2	18	22	2.5
A	*Botryotinia fuckeliana*	PP	16 389	22 334	10 982	58	5	19	2	5	3	2.7
A	*Fusarium oxysporum*	PP	17 735	30 161	9248	67	3	33	0	4	5	
A	*Gibberella zeae*	PP	23 218	38 261	21 355	91	14	75	1	9	16	5.9
A	*Magnaporthe grisea*	PP	14 010	18 795	88 292	86	35	222	31	62	128	7.9
A	*Mycosphaerella graminicola*	PP	10 952	17 661	32 194	83	33	140	9	29	55	6.1
A	*Phaeosphaeria nodorum*	PP	15 983	21 371	15 973	79	9	20	1	2	7	2.4
A	*Sclerotinia sclerotiorum*	PP	14 446	20 240	1844	74	1	2	0	1	0	
B	*Cryptococcus neoformans* B-3501A	HP	6583	15 244	74 724	92	69	900	31	106	229	18.2
B	*Cryptococcus neoformans* JEC21	HP	6604	15 554	74 724	92	70	945	31	120	244	19.9
B	*Coprinopsis cinerea*	NP	13 544	30 180	15 777	84	15	173	4	15	36	8.6
B	*Laccaria bicolor*	NP	18 216	36 757	34 345	87	21	253	18	35	74	5.9
B	*Phanerochaete chrysosporium*	NP	10 048	48 688	12 869	97	18	186	5	21	51	7.7
B	*Ustilago maydis*	PP	6522	4279	39 308	88	50	34	13	14	36	2.3
M	*Rhizopus oryzae*	HP	17 459	40 515	13 313	85	9	26	0	4	11	2.3
											Mean	6.4

Note, for *P. brasiliensis* and *C. neoformans* the same EST data were used for all strains, and hence, the same EST statistics come about. Roche 454 transcript sequences are used for *A. benhamiae*, and classical EST data for all other species. AS rates in the last column are from random sampling.

^a^Taxa are Ascomycota (A), Basidiomycota (B) and Mucoromycotina (M). Yeasts are underlined.

^b^Lifestyle: non-pathogenic (NP), plant pathogenic (PP), human pathogenic (HP).

### 2.2. Transcriptome-genome alignments and splice site conservation

Spliced transcript-genome alignments were built in two steps: ESTs were mapped with Blat^26^ to obtain first rough guide alignments. The best Blat hits were further splice aligned with exalin.^27^ To use SS information as additional input for exalin, we prepared a scoring matrix based on SS consensi from *Neurospora crassa* as suggested by Zhang *et al*. Since SSs are conserved among fungi, this model was used for the analysis of all species. Alignments were filtered for minimal score (20 bits), mismatches (≤10%, no mismatches in 5 nt region of SSs) and minimum length of exons and introns (6 and 40 nt, respectively). Only alignments with SSs from canonical (GT|AG) or well-accepted non-canonical (GC|AG, AT|AC) classes^16^ were considered for further analysis.

SS sequence conservation was calculated as information content per position.^28^ To this end, we extracted the sequence from −4 to +7 nt from the exon–intron boundary, and the region from −4 to +4 nt from the intron–exon boundary. We used the upstream boundary of alternative 3′ SSs and the downstream boundary of alternative 5′ SSs.

### 2.3. Detection of AS

Custom Perl scripts were used to analyse filtered transcript-genome alignments for four AS events: exon skipping (cassette exon), alternative 5′SS and alternative 3′SS selection and intron retention. Using splice positions (genomic starts and ends of exons and introns) we compared the positions between all exons and introns to find overlaps and identify the basic AS types. AS events were predicted based on EST discrepancies only, not on discrepancies between ESTs and annotations. To account for the limited sequence data used in our analysis, one EST was considered sufficient to support an mRNA isoform. Constitutively spliced exons and introns were defined as not having support of AS at a minimum coverage of 10 ESTs.

### 2.4. Random sampling of transcripts and per-gene AS rates

Random sampling was done for each genomic location with *n* ≥ 2 aligned transcripts. We randomly drew a defined number of transcripts and estimated the AS rate. To do so, AS events were assigned to genes based on mapping coordinates, and the number of AS affected genes was divided by the overall number of genes detected by random sampling. Then, we multiplied the AS rates with the number of genes having introns (potential AS candidates) divided by the number of all genes. This yielded whole genome AS rate estimations. This procedure was repeated 20 times to calculate a mean AS rate estimation. The procedure was done with different sampling depths, drawing 2–10 ESTs per locus. Due to a low EST coverage, loci with a higher coverage than 10 ESTs are rare for most analysed species. Thus, to avoid a bias towards highly expressed genes, results from lower sampling depths were kept in sampling repeats with higher sampling depths. That is, sampling depth *i* means to draw at most *i* ESTs from a locus. Pearson's product moment and its corresponding significance test was used to assess the correlation between AS rates and number of mapped ESTs (based on Student's *t*-test, assuming normal distribution of the data, R version 2.12.1^29^). Four species were excluded from correlation analysis because <5% of their annotated genes were covered by the sampled ESTs (Table 1 and Supplementary Table S2): *Aspergillus oryzae*, *Chaeto-mium globosum*, *Fusarium oxysporum*, *Sclerotinia sclerotiorum*.

### 2.5. Functional gene annotations and enrichment statistics

Genes were searched for protein domain motifs using HMMER3^30^ (*e*-value < 0.01) together with the Pfam database (release 24). Alternatively, Pfam domain annotations were downloaded from the Broad Institute (*Fusarium* sp., *Paracoccidioides* sp., *R. oryzae*). Associations between Pfam domains and AS were tested using the following model: per species, all genes that have at least two EST hits are taken into consideration with their Pfam assignment and number of introns. Thereof, all introns are assumed to have an equal, species-specific probability *p* to be alternatively spliced, as averaged from the empirical data. The probability *P*(*g*∈AS) that a gene with *n* introns is alternatively spliced is calculated as *P*(*g*∈ AS) = 1 − (1 − *p*)*^n^*.

Then, the expected number of alternatively spliced genes coding a certain Pfam is calculated by cumulation: Exp(*n*_AS,Pfam_) = Σ*P*(*g_i_* ∈ AS).

The distribution of *n*_AS,Pfam_ was obtained from Monte Carlo simulation of the cumulation terms (*n* = 10^6^). Binomial rather than hypergeometric simulation of *P*(*g_i_* ∈ AS) simplified the calculations and yielded a slightly wider distribution, resulting in conservative estimates of the distribution quantiles. Correction for multiple testing was done using the Bonferroni method.

## 3. Results

### 3.1. Mining of introns and splicing signals

The number of available ESTs per species varies in a wide range (1557–1 040 774). To detect AS, at least two transcripts per locus are needed, i.e. one for each of at least two splicing isoforms. We find that, depending on the species, 0–100% of the annotated introns are overlapped by at least two ESTs (25% on average over all fungi; Supplementary Table S2), and 1–86% of genes are overlapped by at least two ESTs (28% on average; Table 1 and Supplementary Table S2). Per species, 98–100% of the detected introns per species harbour typical SSs (GT|AG), whereas non-canonical SSs (GC|AG, AT|AC) are rare (0–2%), in accordance with a previous study on fungi.^16^ The sets of reliable genomic intron and exon coordinates were subsequently examined for AS events.

### 3.2. Whole genome AS rates

The numbers of detected AS events strongly depend on the numbers of available ESTs (Supplementary Fig. S1a; Pearson correlation coefficient *r* = 0.82, *P*-value 1.8 × 10^−6^). A very high coverage of introns with ESTs, especially when using next generation transcriptome sequencing, can reveal even very rare events that may partly represent splicing noise of the cell. This can lead to overestimation of AS propensity of a species. On the other hand, an uneven genomic distribution of transcripts leads to an under-sampling of the genome-wide splice isoforms. To circumvent these pitfalls, we applied a random sampling strategy, similar to the one of Kim *et al*.^7^ to obtain AS rate estimations that are independent of EST amounts and distributions. We left out species where <5% of multi-exon genes were covered by the sampled ESTs for estimation of whole genome AS rates (last column Supplementary Table S2). For them we do not expect the estimations to be reliable enough. We mapped the AS events that were recovered by random sampling to genomic locations of annotated genes to calculate AS rates per gene. We found that the correlation between these AS rates and the EST numbers is clearly reduced (*r* = 0.16, *P*-value = 0.46, Supplementary Fig. S1b). Thus, random sampling gives AS rate estimates that are comparable between species.

The more ESTs were sampled from a genomic location (sampling depth) the higher is the chance of finding AS events (Supplementary Fig. S2). We decided to sample up to 10 ESTs per locus to reduce the chance of sampling rare events and, thus, overestimation of AS capacities. The reduced gains of AS rates with higher sampling depth support this decision (decreasing slopes of curves in Supplementary Fig. S2). Thus, the following results refer to a sampling depth of 10 ESTs, if not stated differently.

**Figure 1.**
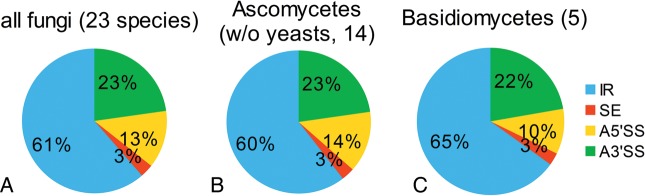
Alternative splice type distribution per taxon from random sampling approach. Pie portions: intron retention (IR), skipped exons (SE), alternative 5′ splice sites (A5′SS) and 3′ splice sites (A3′SS). Only the 23 informative fungi are considered, i.e. those where AS rates could be estimated (cf. Table 1). Only non-yeasts are considered in chart 1B (17 ascomycetes − 3 yeasts = 14).

6.4% of fungal genes are affected by AS when averaging on species level (Table 1 and Supplementary Table S2). Excluding ascomycetous yeasts (*P. stipitis*, *S. cerevisiae* and *S. pombe* 0.26% AS affected genes), the rate is 7.3%. *Coccidioides immitis* and *C. neoformans* show outstanding AS rates of 13 and 18/20%, respectively (strains JEC21 and B-3501A). The relative proportions of the AS types averaged over all species, in the order of frequency are: intron retention 61%, alternative 3′ SSs 23%, alternative 5′ SSs 13% and skipped exons 3% (Fig. 1a). We only took into account strains B-3501A and Pb01 from *C. neoformans* and *P. brasiliensis*, respectively, for mean value calculation.

### 3.3. Validation of retained introns

An alternative explanation for detected retained introns (RIs) would be the presence of unprocessed pre-mRNA in the sequenced samples or a contamination with DNA. First of all, the EST libraries used for this study were all prepared from total RNA and enriched for poly(A)-mRNA. This makes DNA contamination very unlikely. To further validate the detected RIs, we assessed the number of RI-supporting ESTs that have been already processed in the following way. For each species, we counted the number of RIs where at least one EST of the isoform that harbours the RI supports a spliced intron at another EST position. For all species with RIs, between 74 and 100% (average 96%) of those isoforms contain a processed intron (details see Supplementary Table S3). This clearly indicates that most RIs are authentic RNA events.

### 3.4. Correlations of AS rates and genomic features

We calculated the correlations between genome and splicing quantities. We find a strong correlation (*r* = 0.73, *P*-value 8.8 × 10^−5^) between the number of EST-covered introns and the number of RIs across the species. This hints at fungal introns to have a certain chance *per se* to be retained in an alternative manner. In contrast, there is only a slight and barely significant correlation of the extrapolated genome-wide AS numbers with gene numbers (*r* = 0.41, *P* = 0.0502) and with genome size in nucleotides (*r* = 0.53, *P* = 0.009). Further, there is no correlation of the AS rate per gene with gene numbers (*r* = −0.05, *P* = 0.82) nor with genome size (*r* = 0.12, *P* = 0.57). Nonetheless, the small genome sizes (in base pairs and gene numbers) of the yeasts *S. cerevisiae*, *S. pombe* and *P. stipitis* coincide with their clearly reduced AS propensity.

### 3.5. Intron retention is the major AS type in fungi

Intron retention makes up two-thirds of the AS events in the investigated fungi. This also holds for each fungal group separately. We investigated the properties of affected introns and their aberration from constitutively spliced ones. We find that RIs are shorter (89 nt) than constitutively spliced introns (93 nt), on average across all species, though not significant (Mann–Whitney U-test, *P* = 0.211, *n* = 5665/23 268). Neither constitutively spliced nor RIs tend to preserve the reading frame. That is, in both sets, intron lengths are distributed evenly over the three possible remainders of division by three. Constitutively spliced introns: remainder zero, 32%; remainder one, 34%; remainder two, 34%; RIs: 33, 34 and 33%, respectively.

### 3.6. Varying alternative splice propensity is taxon-dependent

**Figure 2.**
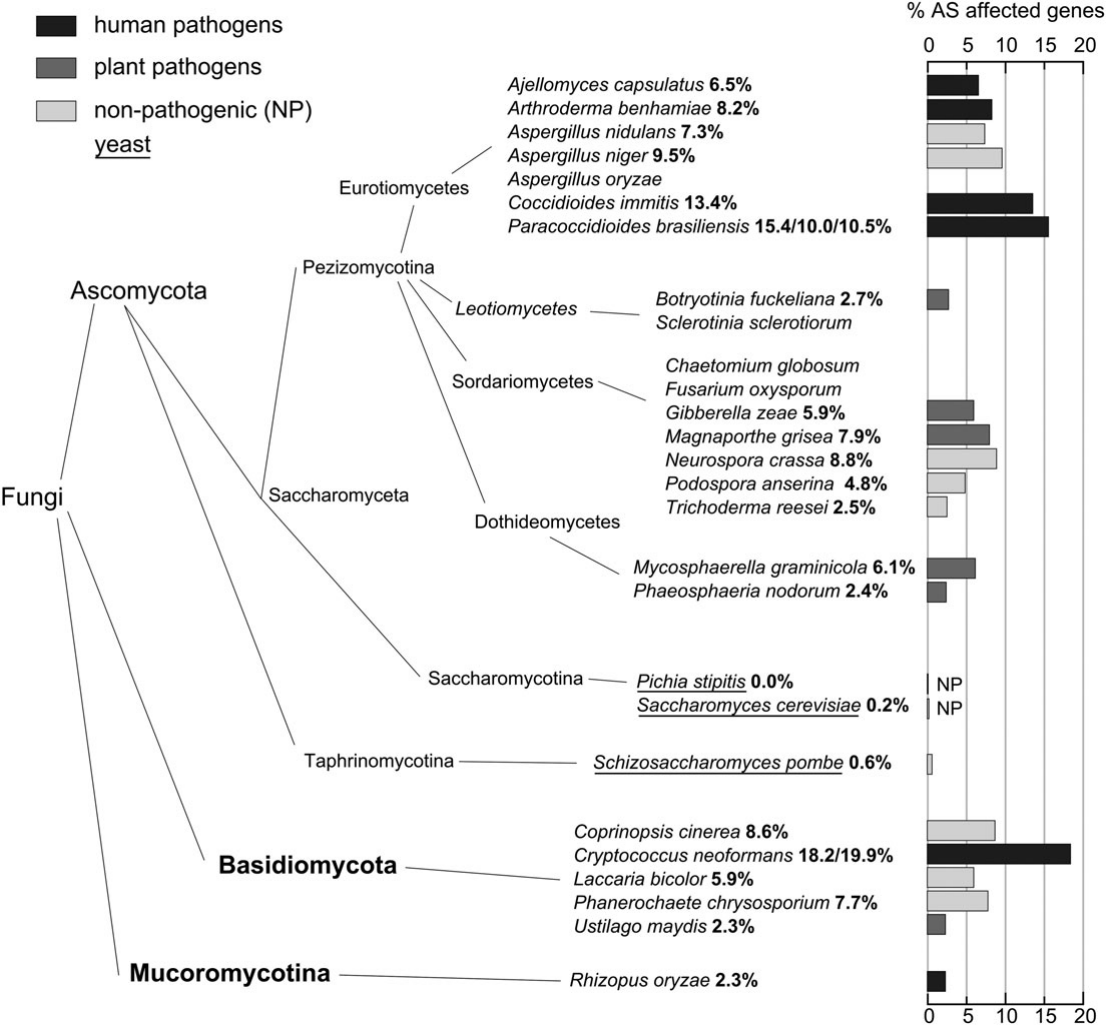
Species tree. This phylogenetic tree shows the evolutionary relationship between the analysed species, based on James *et al*.^31^ Percentages and bars next to the species represent the estimated AS rates per gene. AS rates for each strain are shown in case of species with more than one analysed strain. Species' lifestyles are colour coded: human pathogens, black; plant pathogens, dark gray; non-pathogenic fungi, light gray. Yeasts are underlined.

We summarized and averaged the resampled AS rates into two different fungal taxa (see a species tree in Fig. 2). On average in Basidiomycota more genes are affected by AS (8.6%) than in Ascomycota (7.2% w/o ascomycetous yeasts, Mann–Whitney U-test, not significant, *n* = 5/14). Without the species showing outlying AS rates (*C. immitis*, *P. brasiliensis*, *C. neoformans*), the rates for Basidiomycota (6.1%) and Ascomycota (4.9%) are still different. Basidio-mycota and Ascomycota have very similar AS type proportions, with Basidiomycota showing slightly more RIs and less alternative 5′ SSs (Fig. 1). In both cases RIs make up around two-thirds of all AS events while skipped exons are only marginally present. The ascomycetous yeasts of our study (*P. stipitis*, *S. cerevisiae* and *S. pombe*) show an AS rate of 0.26% on average, which is significantly lower than the rate of the other Ascomycota (Mann–Whitney U-test, *P*-value 0.003, *n* = 3/14). An explanation for this difference may be deviations in structural gene properties that influence splicing. We find that Basidiomycota have on average shorter constitutively spliced introns (86 nt) than Ascomycota (96 nt, Mann–Whitney U-test, *P* < 2.2 × 10^−16^, *n* = 5205/17 936), and also shorter RIs (72 nt vs. 95 nt, *P* < 2.2 × 10^−16^, *n* = 1545/4093). Considering the ascomycetous yeasts separately, they show on average 326-nt long constitutively spliced introns and 132-nt long RIs, though it should be noted that yeast RI data are only based on five introns. In contrast, the one Mucoromycotina (formerly Zygomycota) *R. oryzae* has very short constitutively spliced introns (61 nt) and RIs (54 nt).

To further support the idea of the influence of gene properties on taxon-dependent AS frequencies, we compared the average conservation of SS motifs (Supplementary Fig. S3a and b). Sequence conservation in terms of information content can be considered as a proxy for SS fidelity. We find that ascomycetous retained as well as constitutively spliced introns show higher SS conservation than the corresponding basidiomycetous ones (not significant, Mann–Whitney U-test, all *P* > 0.08). The one Mucoromycotina, *R. oryzae*, has higher SS conservation in both types of introns than the Basidiomycota, yet cannot clearly be distinguished from Ascomycota in this respect. Yeasts show the highest SS conservation. However, the number of sampled yeasts and Basidiomycota are very small so that only 5′SSs of yeast RIs are significantly more highly conserved than 5′SSs of basidiomycetous RIs (*P* = 0.036, *n* = 2 yeasts (yeast *P. stipitis* contributes no RIs), 5 Basidiomycota).

### 3.7. Functional characterization of AS

To study the function of fungal AS we analysed annotated and predicted Pfam domains for all genes and their relations to the AS rate of the gene families. We pooled all data and asked if particular Pfam domains are associated with higher AS rates. In a neutral model, AS is homogeneously distributed over all introns. Based on this model, we calculated the expected fraction of AS-associated genes per Pfam domain and compared it with the observed AS fraction. Together, six significantly AS-enriched Pfam gene families were identified (Supplementary Table S4). Two are ribosomal genes (PF01479, PF01599) and two are genes involved in thiamine biosynthesis (PF09084, PF01946). We note that these gene families show particularly high expression rates (on average, EST coverage is 34, compared with 0.6 for a non-AS-enriched control group). Since ESTs are the primary evidence for AS, and the detection rate of AS increases with EST coverage, it is well possible that the high expression rates alone account for the Pfam-AS association in these gene groups.

Apart from these, the other two significantly AS-enriched Pfam gene families are fungi specific (PF08520, PF12586) with unknown domain function. Remarkably, domain PF12586 occurs only in Cryptococcus. The next Pfam gene family with a known function, though below global significance (*P* = 0.35 with Bonferroni correction), is PF03073 and comprises integral membrane proteins that act as negative regulators of gene expression in response to oxygen or light (Supplementary Table S5).

### 3.8. AS is associated with dimorphic switch and pathogenicity

Comparing AS rates of pathogenic and non-pathogenic fungi, we found interesting aspects: the rate in pathogenic species is higher (7.6%) than in non-pathogenic species (5.1%). Considering only human pathogens, the rate of 10.7% is even more striking, yet the differences are not significant (Mann–Whitney U-test, all *P*-values >0.09, *n* = 11 non-pathogenic, 6 human pathogenic).

The Pfam domain descriptions of the AS affected genes pointed to an involvement in stress response to an altering environment as it occurs during host infection: heat shock proteins, chaperone/chaperonin. These proteins mediate stress response, for example thermo-tolerance in mammalian hosts.^32^ Furthermore, AS-affected genes are often related to availability of copper, which is typical when penetrating human host tissue: multi-copper oxidase and CTR copper transporter family. Glucuronoxylomannan, the predominant capsular polysaccharide in *C. neoformans*, experiences a structural change during dimorphic switching. Thus, the capsule surface changes, which results in a reduced recognition by the host's immune system.^33^ We identified homologues of the proteins involved in the production and modification of glucuronoxylomannan for all investigated fungi via sequence similarity using BLASTP. Four of these proteins do show AS association, namely RIs, two in *C. neoformans* JEC21 and two in *C. neoformans* B-3501A. Three are hypothetical proteins harbouring a glycosyltransferase GTB or CAP59 mtransfer region. Two are annotated as mannosyltransferase 1 (Supple-mentary Table S6). However, the predicted homologues are not significantly enriched in AS association (hypergeometric test, *P* > 0.1).

Another virulence factor is the adaptation of a fungus to the altered environment of the host tissue. Up-regulation of oxidative and heat shock stress associated genes as *tps1*, *hsp30* and *ddr48* in *P. brasiliensis* P01 likely convey to cope with this micro-niche climate.^34^ The identified homologues of these 3 genes are frequently affected by AS in pathogenic fungi (15 cases) and 5 times in non-pathogenic fungi (Supplementary Table S7). Among the Tps1 homologues are genes from *C. neoformans* B-3501A and JEC21, one of *P. anserina* and one of *T. reesei*, an alpha,alpha-trehalose-phosphate synthase Tps1 subunit of *L. bicolor* and a hypothetical protein similar to alpha,alpha-trehalose-phosphate synthase subunit TPS3 of *N. crassa*. Some of the genes are affected by multiple AS events. The AS-associated Hsp30 homologues are three chaperones/small heat shock proteins from *C. immitis*, *L. bicolor* and *U. maydis*. Finally, Ddr48-homologues with AS association are hypothetical/predicted proteins of *C. immitis*, *A. capsulatus*, *C. neoformans* and *M. graminicola*, two of which have a predicted function: ‘similar to potential stress response protein’, and ‘Glycosyltrans-ferase GTB type’. These stress response-related proteins are significantly enriched in AS association (hypergeometric test, *P* = 0.00022).

## 4. Discussion

### 4.1. AS rate estimation

We here presented a comparative genome-wide survey of AS in the fungal kingdom. We based our survey mainly on Sanger-sequenced EST data (one species' ESTs are from 454 sequencing) and corresponding annotated genomes. In current AS studies, often next generation transcriptome sequence data with millions of short ESTs are used. However, though for some fungi, these data are available, the other prerequisite of having a well-annotated genome is rarely fulfilled. According to our results, next generation sequencing technologies with EST lengths of >200 nt (met by Roche 454 as well as Illumina/Solexa platforms) should be well feasible for the detection of the basic AS types in fungi. This is because the read length is clearly longer than the average fungal intron and exon lengths (constitutively spliced introns 93 nt and exons 132 nt).

The alignment of transcript sequences to genomes is currently the most effective way to detect alterations of mature mRNA at a large scale. However, Fox-Walsh and Hertel^9^ argued that every multi-exon gene has a certain AS frequency, and the detection of an alternative isoform is a matter of sensitivity of the method applied. Thus, we here use a random sampling approach similar to the one by Kim *et al*.^7^ This universal normalization approach led to AS rate estimates that are independent of the number and distribution of the ESTs, and thus, are more comparable across species. We found AS events in every of the 27 studied fungi except one (*P. stipitis*), with an average rate of 6.4% of genes. Thus, we suppose that AS is a common phenomenon in the fungal kingdom. *Coccidioides imitis* and *C. neoformans* show outstanding AS rates of 13 and 18%, the latter being about three times more than anticipated in earlier studies.^17,20^ While successively increasing the sampling depth from 2–10, we found that the relative proportions of the AS rates between most species remain constant (Supplementary Fig. S2). This underpins the reliability of our normalization method. Because many loci have a lower EST coverage than the sampling depth of 10, our analysis yielded rather conservative estimates. It is likely that with deep transcriptome sequencing more fungal AS events will be found. Even when excluding very rare events, this may elevate the AS rates. This trend was seen for human and other mammals already,^8^ and can be supported by the finding that for *A. benhamiae* and *N. crassa*, both of which have high EST coverage, the AS rates clearly kept rising at higher sampling depths (Supplementary Fig. S2), opposed to most of the other species.

Finally, in a recent study on fission yeasts, 433 AS events in overall 5144 genes were found in *S. pombe*.^35^ While considering scaling effects due to sequencing depth, our results agree well with these findings in that the AS rate is very low compared with that in non-yeast Ascomycota (see Supplementary Calculation S1). This validates the comparability of our normalized AS rate results.

### 4.2. Fungal introns have an innate propensity to be retained

We found that the trend of relative AS type distribution was the same in all the investigated fungal species. Intron retention made up the most prevalent of the investigated types (61% of the events). Contrarily, skipped exons were very rare (3%) and alternative 3′ (23%) and 5′ SSs (13%) comprised a third of the events. These results are in general agreement with previous findings on fungal AS^15^ and are similar to trends in plants.^7,15^ In contrast, skipped exons are more common than RIs in invertebrates and even more frequent in vertebrates.^7^

The more introns a species genome harbours the more splicing needs to take place. The question is whether this also increases the chance to have alternatively spliced introns *per se*. Indeed, we found a strong correlation of genome-wide intron numbers and numbers of RIs. Thus, fungal introns seem to have an innate chance to be alternatively spliced. Similarly, Irimia *et al*.^17^ found a correspondence between AS and intron number per gene in 12 eukaryotes.

We found that fungal RIs are shorter than constitutively spliced introns. Also, on the species level, there is a correspondence between intron lengths and their propensity to be alternatively spliced. Together, this hints at an involvement of the intron length in the recognition of introns. The intron definition mechanism is a model proposed to explain this same effect in plants. Splicing factors bind to the recognition sites on the RNA, and ‘bridge’ across the intron by mutual binding. Thus, failed recognition of one SS typically results in intron retention.^15^ This is in contrast to metazoan splicing, where splicing factors are assumed to form stable complexes across exons (exon definition mechanism) and where failed SS recognition typically results in exon skipping. It explains why metazoan introns tend to be much longer (e.g. 3413 nt in human^36^) but are rarely retained. Thus, we propose that the intron definition mechanism is prevalent in fungi similar to plants.

Finally, there is a hypothesis that connects SS conservation with splicing propensity, saying that strict adherence to the SS motif promotes the splicing machinery to bind more reliably to the SS and thus decreases the chance of AS.^37^ McGuire *et al*.^15^ find weaker (i.e., less conserved) SSs at RIs compared with constitutively spliced introns in all their investigated species. Here, when comparing introns (both retained and normally spliced ones) between the taxa, we find that higher SS conservation correlates with lower AS rates, which supports the hypothesis.

### 4.3. Fungal RIs are authentic and likely trigger nonsense-mediated mRNA decay

There is a debate if RIs are authentic AS events or represent incompletely spliced pre-mRNA. Contamination with genomic DNA is very unlikely since the construction of EST libraries relies on affinity-based poly(A)+ mRNA enrichment. From the analysis of fungal RIs, we found no tendency to preserve the reading frame, similar to results on non-fungal species in a previous study.^15^ This may support the hypothesis of spurious intron retention. However, we have several arguments against it. For the majority of EST libraries analysed here, cDNA was produced by poly(A)-tail capture, ensuring that ESTs derive from fully transcribed mRNAs. The current consensus is that intron splicing occurs predominantly cotranscriptionally,^38^ corroborated by findings that the nascent mRNA can recruit multiple spliceosomes simultaneously.^39^ Though the exact kinetics of RNA processing and export are unknwon, intron splicing is likely finished shortly after transcription. This supports the hypothesis that if a detected multi-intron mRNA was spliced at one intron, it has already been spliced at the other introns, too. In fact, averaged over all species, 96% of the transcript isoforms that support an RI contain a processed intron at another position, as was similarly reported for RIs in *Arabidopsis thaliana*.^40^ In these cases, the completed splicing of co-transcribed introns indicates that the molecules have passed spliceosomal processing and that RIs likely represent authentic events on mRNA. However, it is possible that RI-containing mRNAs had not left the nucleus, awaiting a later processing cycle or degradation. Nevertheless, even if this is true, these cases illustrate inherent differences in splicing efficiency.

It was argued that despite a weak selection for coding potential, splice variants having RIs unlikely yield functional proteins.^15^ While we suppose that most RIs are authentic AS events, the isoforms with a frame-shifting RI unlikely yield productive, protein-coding mRNAs. However, we hypothesize that fungal RIs may in part be a means for post-transcriptional regulation via nonsense-mediated mRNA decay (NMD), in which transcripts containing premature termination codons (PTCs) are degraded.^41^ This is because RI sequences with frame shifts probably introduce PTCs (15 randomly drawn triplets pose a chance of >50% to contain a stop codon). Most of the NMD-related components^41^ are conserved in most of the fungi present in NCBI's HomoloGene database (Supplementary Table S8). *Saccharomyces cerevisiae* has an NMD machinery which is, however, not essential. Most RIs of the yeast *Yarrowia lipolytica*, contain PTCs and there is evidence that corresponding RNA is degraded by NMD.^42^ Finally, first evidence for functional NMD were found in *N. crassa*.^43^ As long as experimental data for a functional relevance of RIs are missing, we note that RIs qualify as mediators for a splicing-dependent mechanism of gene expression regulation, based on structure as well as on statistical association with functional categories (see below).

### 4.4. Does AS facilitate multi-cellular complexity?

The complexity of the (multi-)cellular structure has long since been an important feature to classify fungi into sub-taxa.^44^ Typical instances of diverse complexity are, being yeast or mold, and characteristics of sexual structures. There are predominantly single-celled yeasts, namely *S. pombe*, *S. cerevisiae* and *P. stipitis*, within the phylum of the Ascomycota, whose most complex yeast form is a four-spore ascus. The Mucoromycotina *R. oryzae* forms simple zygospores during sexual reproduction, but differentiated multi-cellular sporangia for asexual reproduction. Filamentous Ascomycetes produce more complex thalli, as, e.g. ascocarps (apothecium, cleistothecium, perithecium). Finally, Basidio-mycota, probably the most recent ‘crown group’ of fungi, develop complex fruiting bodies.^44^ We here find that the average AS rate of the mentioned taxa correlates with this order of complexity: *Saccharomycotina* and *Taphrinomycotina* (0.26% per-gene AS rate), Mucoro-mycotina (2.3%), Pezizomycotina (7.2%, Ascomycota excluding yeasts) and Basidiomycota (8.6%). We speculate that AS contributes to multi-cellular complexity of the fungi.

We find that the fungi with the smallest genomes show nearly no AS. These are the ascomycetous yeasts *S. cerevisiae*, *S. pombe* and *P. stipitis*. This is consistent with an earlier study on *S. cerevisiae* and *S. pombe*.^17^ A major reason for this is probably the reduced proportion of intron-containing genes, e.g. 5% of *S. cerevisiae* genes vs. 86% in *C. immitis*, since Hemiascomycetes (Saccharomycetes) experienced intron loss during the course of evolution.^45^ However, from a certain genome size on, neither the AS rate nor the absolute AS number show any correlation. And, to an extreme, *C. neoformans* has only ca. 6600 genes but the highest found AS rate (18%).

The composition of the splicing machinery can give another perspective in understanding the differences in AS capability. The core components of the spliceosome, i.e. the five snRNPs and essential dynamic factors like Prp8 or Slu7, are generally conserved in eukaryotes. However, the small subunit of U2AF (U2AF35 in human), involved in recognition of the 3′SS, is absent in *S. cerevisiae*. The family of serine/arginine-rich (SR) proteins comprise many known splicing regulators, and it was proposed that a higher SR protein diversity increases the AS complexity.^46^ Our results do support this hypothesis: among yeasts, which have the lowest AS rates, *S. cerevisiae* has no SR proteins, only an SR-like homologue Npl3,^47^ and *S. pombe* has only two SR proteins.^48^ On the other hand, many of the other species of our study were found to have many SR and SR-related proteins,^49^ in accordance with their higher AS rates.

We used Pfam domain annotations to analyse the possible functional associations of AS. The most significantly AS-enriched Pfam-coding gene families are ribosomal or do function in thiamine biosynthesis. However, these findings should be taken with caution since the expression level (i.e. EST coverage) is ∼50-fold higher than average. Other AS-enriched gene families with moderate gene expression levels do code for fungi-specific protein domains of unknown function. This may indicate that AS is associated with enhanced evolutionary dynamics in these gene families, consistent with a supportive role of AS in gene evolution.^50^

Taking together the relatively low fraction of AS-associated gene families and the gene expression bias among the few candidates, we conclude that a homogenous distribution model is currently a sufficient explanation for the occurrence of AS among the EST-covered genes. However, we anticipate that increasing EST sequencing depths, and a saturation of a major fraction of genes, will allow more detailed insights into the functional association of AS in fungi.

Both, the elevated AS rates and the greater amount of splicing regulators of the more complex fungi suggest the hypothesis that AS may facilitate multi-cellular complexity. Furthermore, we found that AS is involved in another elaborate trait of certain fungi, namely virulence.

### 4.5. AS likely regulates virulence of pathogenic fungi

A first hint of AS involvement in pathogenicity was given by mere comparison of average AS rates. Human pathogenic fungi show on average a twice as high AS rate (10.7%) than non-pathogenic fungi (5.1%, neither plant nor human pathogenic). This is corroborated by the keywords of AS-associated Pfam domains which indicate enrichment for stress response functions. Moreover, a direct search for homologues of *P. brasiliensis*' *tps1*, *hsp30* and *ddr48* genes that convey cell rescue of this fungus while facing oxidative and heat shock stress in the human body,^34^ yielded many AS-associated genes in human and plant pathogenic fungi. Hence, it is likely that AS is involved in gene expression regulation during the adaptation to the environmental conditions in the host.

The dimorphic switch is another virulence factor, and a key of persistent virulence.^33^ During host penetration, a fungus can either switch to filamentous growth (e.g. *C. albicans*, *A. fumigatus*), or switch from filamentous to uni-cellular growth (e.g. *P. brasiliensis* Pb01, *C. immitis*). The dimorphic switch is only poorly understood. However, several contributing compounds have been identified. *Cryptococcus neoformans*' glucuronoxylomannan (GMX), a capsular polysaccharide, is crucial for switching, as it alters the capsule surface. This increases the resistance against host immune system by hampering antibody and complement mediated phagocytosis.^33^ We found two homologues of GMX production and modification proteins in *C. neoformans* (in B-3501A and JEC21), each containing an RI. Of the 19 predicted homologues of *tps1*, *hsp30*, *ddr48* and GMX-related genes, 5 have AS association in four non-pathogenic fungi (Supplementary Tables S6 and S7).

An association of AS with pathogenicity has been found in former studies already. The *UrRm75* gene in *U. maydis*, involved in dimorphism and virulence, contains four introns and has an alternative 3′SS.^51^ A putative heat shock protein and a putative alpha, alpha-trehalose-phosphate synthase (both stress response-associated) were predicted to be affected by AS in *C. neoformans*.^20^ Transcripts of cryptococcal intersectin 1 undergo AS and its disruption affects the production of several virulence factors in *C. neoformans*.^52^ In many fungi, Ste12-like transcription factors play essential roles in invasive growth and pseudohyphal development, and their gene transcripts are affected by AS within a conserved exon–intron structure.^53^ Summar-izing, gene regulation via AS likely facilitates virulence of pathogenic fungi on various levels.

## Funding

This work was funded by the Friedrich-Schiller-University Jena and the Jena School for Microbial Communication (JSMC).

## Supplementary Material

Supplementary Data
