# Supplementary material for: Fungal Alternative Splicing is Associated with Multicellular Complexity and Virulence: A Genome-Wide Multi-Species Study
Source: DNA Res. 2013 Oct 11;21(1):27–39. doi: 10.1093/dnares/dst038 (PMC3925392; doi:10.1093/dnares/dst038)
Supplement: Supplementary Data [file supp_21_1_27__index.html]

Fungal Alternative Splicing is Associated with Multicellular Complexity and Virulence: A Genome-Wide Multi-Species Study — Fungal Alternative Splicing is Associated with Multicellular Complexity and Virulence: A Genome-Wide Multi-Species Study — Supplementary Data 

# Fungal Alternative Splicing is Associated with Multicellular Complexity and Virulence: A Genome-Wide Multi-Species Study

## Supplementary Data

Supplementary Data

**Files in this Data Supplement:**

- Supplementary Data - Pdf file
