# Supplementary material for: Fungal Alternative Splicing is Associated with Multicellular Complexity and Virulence: A Genome-Wide Multi-Species Study
Source: DNA Res. 2013 Oct 11;21(1):27–39. doi: 10.1093/dnares/dst038 (PMC3925392; doi:10.1093/dnares/dst038)
Supplement: Supplementary Data [file supp_dst038_dst038supp.pdf]

SUPPLEMENTARY MATERIAL

Supplementary Tables

Table S1: **Phylum, anamorphic and teleomorphic synonyms and relevance of the investigated fungi.**

phylum	species	anamorph / teleomorph	relevance
Ascomycota	*Ajellomyces capsulatus*	*Histoplasma capsulatum* (anamorph)	systemic mycosis (histoplasmosis) in humans (1)
Ascomycota	*Arthroderma benhamiae*	*Trichophyton erinacei* (anamorph)	superficial mycoses of keratinized host structures in humans and animals (2)
Ascomycota	*Aspergillus nidulans*	*Emericella nidulans* (teleomorph)	important model organism in genetics (3)
Ascomycota	*Aspergillus niger*		production of citric acid and numerous commercial enzymes (3)
Ascomycota	*Aspergillus oryzae*		food fermentation (3)
Ascomycota	*Botryotinia fuckeliana*	*Botrytis cinerea* (anamorph)	plant pathogen, causes gray mold disease on more than 200 host species (4)
Ascomycota	*Chaetomium globosum*		decomposition of cellulose-rich materials, causes skin and nail infections in humans, rarely cerebral and systemic infections, can act as allergen[1]
Ascomycota	*Coccidioides immitis*		causes systemic mycosis, coccidioidomycosis (Valley Fever) (5)
Ascomycota	*Fusarium oxysporum*		plant pathogen with broad host range, e.g. vegetables, cotton, banana, date and oil palm (6)
Ascomycota	*Gibberella zeae*	*Fusarium graminearum* (anamorph)	plant pathogen, head blight in wheat and other small grains, caused with other fusarium species more than 1 bn. US$ damage in US agriculture in 1993 (7)
Ascomycota	*Magnaporthe grisea*	*Pyricularia grisea* (anamorph)	plant pathogen, causes rice blast (8)
Ascomycota	*Mycosphaerella graminicola*	*Septoria tritici* (anamorph)	major wheat pathogen worldwide, causes leaf blotch disease (9)
Ascomycota	*Neurospora crassa*	*Chrysonilia crassa* (anamorph)	non-pathogenic model organism for biological research[2]
Ascomycota	*Paracoccidioides brasiliensis* Pb01/Pb03/Pb18		human pathogen, causes paracoccidioidomycosis (South American blastomycosis) (10)
Ascomycota	*Phaeosphaeria nodorum*	*Stagonospora nodorum* (anamorph)	plant pathogen, causes leaf blotch and glume blotch diseases on wheat (11)
Ascomycota	*Pichia stipitis* (yeast)		cellulose fermentation to produce ethanol (12)

[1] www.broadinstitute.de

[2] http://www.nih.gov/science/models/neurospora/

Ascomycota	*Podospora anserina*		non-pathogenic model organism (13)
Ascomycota	*Saccharomyces cerevisiae* (yeast)		budding yeast, food fermentation, important model organism in biology
Ascomycota	*Schizosaccharomyces pombe* (yeast)		fission yeast, non-pathogenic model organism in biology (14), beer brewing
Ascomycota	*Sclerotinia sclerotiorum*		necrotrophic plant pathogen (white mold), requires senescent tissues to establish an infection (15)
Ascomycota	*Trichoderma reesei*	*Hypocrea jecorina* (teleomorph)	non-pathogenic model organism, industrial production of enzymes, food and feed additives (16)
Basidiomycota	*Coprinopsis cinerea*	*Hormographiella aspergillata* (anamorph)	edible mushroom (gray shag) (17)
Basidiomycota	*Filobasidiella neoformans* B-3501A/JEC21	*Cryptococcus neoformans* (anamorph)	human and animal pathogen (18)
Basidiomycota	*Laccaria bicolor*		edible symbiotic mushroom living on plant roots (19)
Basidiomycota	*Phanerochaete chrysosporium*		production of lignin-degrading peroxidases (20)
Basidiomycota	*Ustilago maydis*		plant pathogen, causes corn smut in maize, important model organism (21)
Mucoromycotina formerly *Zygomycota*	*Rhizopus oryzae*		opportunistic human pathogen, causes zygomycosis (22), industrial production of lipases (23)

Table S2: **Annotation, EST mapping and alternative splicing data of the studied species.**

taxon	species	lifestyle[1]	annotated genes	annotated introns	number of available reads	average read length	% filtered and mapped reads	% introns covered with ≥ 2 reads	% genes covered with ≥ 2 reads	retained introns	skipped exons	alternative 5' intron ends	alternative 3' intron ends	% genes w. any type of AS	% genes w. intron retention	% genes w. cassette exon	% genes w. altern. 5' splice site	% genes w. altern. 3' splice site	detected multi-exon genes	% detected multi-exon genes
Ascomycota	*Ajellomyces capsulatus*	HP	9314	16275	26389	401	55	7	11	51	2	5	15	6.5	5.0	0.3	0.5	1.5	704	8
Ascomycota	*Arthroderma benhamiae*	HP	7984	10332	1040774	297	86	100	86	1381	68	292	445	8.2	5.7	0.2	1.4	2.3	7251	100
Ascomycota	*Chaetomium globosum*	HP	11048	17396	1557	425	34	0	1	1	0	0	0							
Ascomycota	*Coccidioides immitis*	HP	10440	17815	62729	758	93	48	49	664	18	152	225	13.4	9.4	0.3	2.6	3.7	4270	48
Ascomycota	*Paracoccidioides brasiliensis* Pb01	HP	9132	28179	41463	560	75	10	33	235	23	67	110	15.4	9.2	0.9	3.0	4.9	1757	22
Ascomycota	*Paracoccidioides brasiliensis* Pb03	HP	7875	19575	41463	560	71	12	35	134	16	31	52	10.0	6.3	0.7	1.6	2.7	1531	23
Ascomycota	*Paracoccidioides brasiliensis* Pb18	HP	8741	24498	41463	560	71	10	32	134	15	31	52	10.5	6.6	0.7	1.5	2.8	1534	20
Ascomycota	*Aspergillus nidulans*	NP	9541	16797	16848	411	89	8	15	81	1	11	14	7.3	6.0	0.1	0.9	1.2	801	10
Ascomycota	*Aspergillus niger*	NP	10597	17668	46938	634	91	32	28	323	7	37	43	9.5	7.6	0.2	1.1	1.3	2686	30
Ascomycota	*Aspergillus oryzae*	NP	12823	20916	9051	729	94	1	9	70	2	5	12							
Ascomycota	*Neurospora crassa*	NP	9841	14323	277147	675	83	50	52	511	57	128	164	8.8	5.5	0.5	1.6	2.3	4052	50
Ascomycota	*Pichia stipitis* (yeast)	NP	5807	2580	19621	762	95	5	21	0	0	0	0	0.0	0.0	0.0	0.0	0.0	138	8
Ascomycota	*Podospora anserina*	NP	10257	11261	51862	859	92	44	30	194	5	43	83	4.8	3.1	0.1	0.8	1.4	2815	40
Ascomycota	*Saccharomyces cerevisiae* (yeast)	NP	5781	332	34915	509	97	55	41	2	0	2	7	0.18	0.05	0.00	0.03	0.11	223	67
Ascomycota	*Schizosaccharomyces pombe* (yeast)	NP	5073	3878	8123	262	78	5	10	3	0	0	0	0.6	0.6	0.0	0.0	0.0	139	6
Ascomycota	*Trichoderma reesei*	NP	9143	18802	44964	691	76	26	40	66	2	18	22	2.5	1.6	0.1	0.6	0.6	2602	35
Ascomycota	*Botryotinia fuckeliana*	PP	16389	22334	10982	856	58	5	5	19	2	5	3	2.7	1.8	0.2	0.6	0.2	580	5
Ascomycota	*Fusarium oxysporum*	PP	17735	30161	9248	408	67	2	3	33	0	4	5							
Ascomycota	*Gibberella zeae*	PP	23218	38261	21355	545	91	5	14	75	1	9	16	5.9	4.7	0.1	0.6	0.9	1091	6
Ascomycota	*Magnaporthe grisea*	PP	14010	18795	88292	691	86	26	35	222	31	62	128	7.9	4.6	0.4	1.3	2.7	2994	28
Ascomycota	*Mycosphaerella graminicola*	PP	10952	17661	32194	705	83	20	33	140	9	29	55	6.1	3.5	0.2	0.9	1.8	2004	26
Ascomycota	*Phaeosphaeria nodorum*	PP	15983	21371	15973	536	79	8	9	20	1	2	7	2.4	1.6	0.1	0.2	0.7	896	7
Ascomycota	*Sclerotinia sclerotiorum*	PP	14446	20240	1844	681	74	1	1	2	0	1	0							
Basidiomycota	*Cryptococcus neoformans* B-3501A	HP	6583	15244	74724	613	92	100	69	900	31	106	229	18.2	14.0	0.6	2.2	4.7	3985	62
Basidiomycota	*Cryptococcus neoformans* JEC21	HP	6604	15554	74724	613	92	100	70	945	31	120	244	19.9	15.2	0.6	2.8	5.3	3923	60
Basidiomycota	*Coprinopsis cinerea*	NP	13544	30180	15777	717	84	19	15	173	4	15	36	8.6	6.8	0.2	0.7	1.9	1694	14
Basidiomycota	*Laccaria bicolor*	NP	18216	36757	34345	566	87	26	21	253	18	35	74	5.9	4.4	0.1	0.7	1.4	3686	23
Basidiomycota	*Phanerochaete chrysosporium*	NP	10048	48688	12869	707	97	12	18	186	5	21	51	7.7	5.7	0.2	1.0	1.9	1818	21
Basidiomycota	*Ustilago maydis*	PP	6522	4279	39308	457	88	23	50	34	13	14	36	2.3	0.9	0.3	0.4	1.1	872	35
Mucoromycotina	*Rhizopus oryzae*	HP	17459	40515	13313	519	85	6	9	26	0	4	11	2.3	0.9	0.3	0.4	1.1	1160	9
													**mean**	**6.4**	**4.4**	**0.2**	**0.9**	**1.6**	**2096**	**30**

Note, for *P. brasiliensis* and *C. neoformans* the same EST data was used for all strains, and hence, the same EST statistics come about. 454 transcript sequences are used for *A. benhamiae*, and classical EST data for all other species.

[1]Lifestyle: non-pathogenic (NP), plant pathogenic (PP), human pathogenic (HP).

Table S3: **Results of intron retention validation.** The third column shows numbers of intron retention events where at least one read that contains the retained intron has been spliced at another position.

species	retained introns	validated retained introns	% validated
Ajellomyces capsulatus	51	47	92.2
Arthroderma benhamiae	1381	1368	99.1
Aspergillus nidulans	81	78	96.3
Aspergillus niger	323	321	99.4
Aspergillus oryzae	70	67	95.7
Botryotinia fuckeliana	19	14	73.7
Chaetomium globosum	1	1	100
Coccidioides immitis	664	661	99.5
Coprinopsis cinerea	173	171	98.8
Filobasidiella neoformans B-3501A	900	875	97.2
Filobasidiella neoformans JEC21	945	925	97.9
Fusarium oxysporum	33	30	90.9
Gibberella zeae	75	72	96
Laccaria bicolor	253	249	98.4
Magnaporthe grisea	222	211	95
Mycosphaerella graminicola	140	134	95.7
Neurospora crassa	511	491	96.1
Paracoccidioides brasiliensis Pb01	235	208	88.5
Paracoccidioides brasiliensis Pb03	134	117	87.3
Paracoccidioides brasiliensis Pb18	134	121	90.3
Penicillium marneffei	1	1	100
Phaeosphaeria nodorum	20	19	95
Phanerochaete chrysosporium	186	184	98.9
Podospora anserina	194	193	99.5
Rhizopus oryzae	26	25	96.2
Saccharomyces cerevisiae (yeast)	2	2	100
Schizosaccharomyces pombe (yeast)	3	3	100
Sclerotinia sclerotiorum	2	2	100
Trichoderma reesei	66	63	95.5
Ustilago maydis	34	34	100
		**mean**	**95.77**
		**min**	**73.7**
		**max**	**100**

Table S4: **Pfam domains positively associated with alternative splicing.**

Pfam accession	Pfam description	P value*	Avg. EST
PF01479	Ribosomal S4 domain	< 0.0007	21.8
PF09084	NMT1/THI5 like; involved in thiamine biosynthesis	0.0014	37.1
PF08520	fungal protein of unknown function (DUF)	0.0048	10.5
PF01946	Thi4 family; involved in thiamine biosynthesis	0.0089	30.3
PF01599	Ribosomal protein S27a	0.015	47.1
PF12586	protein of unknown function (DUF), Cryptococcus	0.045	2.0

* P-values include the Bonferroni correction

Table S5: **Pfam domains slightly positively associated with alternative splicing.**

Pfam accession	Pfam description	P value*	Avg. EST
PF01248	Ribosomal protein L7Ae / L30e / S12e / Gadd45 family	0.069	8.0
PF00163	Ribosomal protein S4 / S9 N-terminal domain	0.12	27.6
PF00900	Ribosomal family S4e	0.28	20.7
PF03073	TspO/MBR family; integral membrane protein that acts as a negative regulator of gene expression in response to oxygen/light	0.35	2.8
PF02453	Reticulon, know as neuroendocrine-specific protein (NSP), associated with the endoplasmic reticulum	0.42	7.5
PF00428	60S acidic ribosomal protein	0.42	13.4

* P-values include the Bonferroni correction

Table S6: **Glucuronoxylomannan-related genes affected by AS.** Sequences were downloaded from NCBI's protein database using the search "(glucuronoxylomannan) AND "fungi"[porgn:txid4751]".

species	protein ID	definition	note
Cryptococcus neoformans B-3501A	134108310	hypothetical protein CNBB3380	"Glycosyltransferase_GTB_type"
Cryptococcus neoformans JEC21	58263500	hypothetical protein	"Glycosyltransferase_GTB_type"
Cryptococcus neoformans B-3501A	134107349	hypothetical protein CNBA6810	"CAP59_mtransfer", "Cryptococcal mannosyltransferase 1; pfam11735"
Cryptococcus neoformans JEC21	58259209	capsular associated protein	"CAP59_mtransfer", "Cryptococcal mannosyltransferase 1; pfam11735"

Table S7: **AS affected genes involved in stress response.** Relation is based on blast similarity to *P. brasiliensis* Pb01 genes TPS1 (NCBI accession EEH35656), HSP30 (EEH37950), and DDR48 (EEH33596).

species	protein ID	definition	note
**TPS1-relation**			
Cryptococcus neoformans B-3501A	134108248	hypothetical protein CNBB3070	"Glycosyltransferase_GTB_type", "GT1_TPS", "Trehalose-6-Phosphate Synthase (TPS)
Cryptococcus neoformans B-3501A	134116029	hypothetical protein CNBI3400	"Glycosyltransferase_GTB_type", "GT1_TPS", "Trehalose-6-Phosphate Synthase (TPS)
Cryptococcus neoformans JEC21	58264051	trehalose-phosphatase	"Glycosyltransferase_GTB_type", "GT1_TPS", "Trehalose-6-Phosphate Synthase (TPS)
Cryptococcus neoformans JEC21	58270702	alpha,alpha-trehalose-phosphate synthase (UDP-forming)	"Glycosyltransferase_GTB_type", "GT1_TPS", "Trehalose-6-Phosphate Synthase (TPS)"
Laccaria bicolor	170098941	alpha,alpha-trehalose-phosphate synthase TPS1 subunit	"alpha,alpha-trehalose-phosphate synthase TPS1 subunit", Glycosyltransferase family 20; pfam00982"
Neurospora crassa	164428605	hypothetical protein NCU00793	"hypothetical protein", "similar to alpha,alpha-trehalose phosphate synthase subunit TPS3", "Glyco_transf_20"
Podospora anserina	171695706	hypothetical protein	"Glyco_transf_20", "GT1_TPS", "Trehalose-6-Phosphate Synthase (TPS)
Trichoderma reesei	48707	alpha,alpha-trehalose-phosphate synthase	catalyzes UDP-glucose + D-glucose-6-phosphate = UDP + alpha,alpha-trehalose-6-phosphate
**HSP30-relation**			
Coccidioides immitis	119194749	30 kDa heat shock protein	"IbpA", "Molecular chaperone (small heat shock protein)
Laccaria bicolor	170101017	predicted protein	"ACD_sHsps-like", "Alpha-crystallin domain (ACD) of alpha-crystallin-type small(s) heat shock proteins (Hsps)
Ustilago maydis	71019595	hypothetical protein UM03881.1	"IbpA", Molecular chaperone (small heat shock protein)
**DDR48-relation**			
Ajellomyces capsulatus	154277766	hypothetical protein HCAG_05184	"similar to potential stress response protein", "PTZ00110", "helicase; Provisional"
Coccidioides immitis	119192856	predicted protein	"hypothetical protein"
Cryptococcus neoformans B-3501A	134108310	hypothetical protein CNBB3380	"Glycosyltransferase_GTB_type"
Mycosphaerella graminicola	103686	-	-

Table S8: **NMD-related components and their homologs in NCBI HomoloGene database.** Note, HomoloGene contains data of only six fungi (*S. cerevisiae*, *K. lactis*, *S. pombe*, *M. oryzae*, *N. crassa*, *E. gossypii*), hence the limited coverage of species.

NMD factor	Homolo-Gene ID	gene	description	species
**UPF1**	2185	NAM7	Nam7p	*S.cerevisiae*
		KLLA0B06435g	hypothetical protein	*K.lactis*
		upf1	ATP-dependent RNA helicase Upf1	*S.pombe*
		MGG_00976	regulator-nonsense transcripts 1	*M.oryzae*
		NCU04242	ATP-dependent helicase NAM7	*N.crassa*
		AGOS_ABR022C	ABR022Cp	*E.gossypii*
**UPF2**	6101	KLLA0D13156g	hypothetical protein	*K. lactis*
		NMD2	Nmd2p	*S. cerevisiae*
		upf2	nonsense-mediated decay protein Upf...	*S. pombe*
		MGG_06063	nonsense-mediated mRNA decay factor	*M. oryzae*
		NCU05267	hypothetical protein	*N. crassa*
**UPF3**	11307	SPAC13G7.03	hypothetical protein	*S. pombe*
	39098	UPF3	Upf3p	*S. cerevisiae*
		KLLA0D03718g	hypothetical protein	*K. lactis*
		AGOS_AER204W	*AER204Wp*	*E.gossypii*
	126047	MGG_03912	hypothetical protein	*M. oryzae*
		NCU03435	hypothetical protein	*N. crassa*
**CBP80**	68864	STO1	Sto1p	*S. cerevisiae*
		KLLA0F17523g	hypothetical protein	*K. lactis*
		AGOS_AFR218W	AFR218Wp	*E. gossypii*
		SPAC6G10.07	nuclear cap-binding complex large s...	*S. pombe*
		MGG_12123	cap binding protein	*M. oryzae*
		NCU04187	hypothetical protein	*N. crassa*
**CBP20**	103828	CBC2	Cbc2p	*S. cerevisiae*
		KLLA0B10472g	hypothetical protein	*K. lactis*
		AGOS_AFL050W	AFL050Wp	*E. gossypii*
		SPBC13A2.01c	nuclear cap-binding complex small s...	*S. pombe*
		MGG_06296	nuclear cap-binding protein subunit...	*M. oryzae*
		NCU00210	nuclear cap binding protein subunit...	*N. crassa*
**Y14**	3744	SPAC23A1.09	RNA-binding protein	*S. pombe*
		MGG_03740	RNA-binding protein 8A	*M. oryzae*
		NCU03226	hypothetical protein	*N. crassa*
**BTZ**	127412	MGG_00982	hypothetical protein	*M. oryzae*
		NCU04270	hypothetical protein	*N. crassa*
**eIF4AIII**	5602	FAL1	Fal1p	*S. cerevisiae*
		KLLA0A10659g	hypothetical protein	*K. lactis*
		AGOS_AER408W	AER408Wp	*E. gossypii*
		SPAC1F5.10	ATP-dependent RNA helicase, eIF4A r...	*S. pombe*
		MGG_04885	ATP-dependent RNA helicase fal-1	*M. oryzae*
		NCU01234	eukaryotic initiation factor 4A-12	*N. crassa*
	127412	MGG_00982	hypothetical protein	*M. oryzae*
		NCU04270	hypothetical protein	*N. crassa*
**MAGOH**	56794	SPBC3B9.08c	protein mago nashi	*S. pombe*
		MGG_06859	mago nashi like 2	*M. oryzae*
		NCU04405	mago nashi protein	*N. crassa*

**SMG1**	44191	smg1	Sm snRNP core protein Smg1	*S. pombe*
		MGG_10740	sm snRNP core protein Smg1	*M. oryzae*
		NCU09880	hypothetical protein	*N. crassa*
**PYM**	no entry for fungi			
**hNAG**	no entry for fungi			
**DHX34**	no entry for fungi			

Supplementary Calculation S1

We downloaded all reads of *S. pombe* of the study on fission yeasts (24) from NCBI's short read archive. We converted the reads to fastq format with fastq-dump 2.0.5 using standard parameters (25). To estimate the number of useful reads we filtered them with the "lite" version of PRINSEQ (26) with the following strict filter settings[3]:

```
prinseq-lite.pl -fastq in.fastq -trim_qual_left 25 -trim_qual_right 25 -min_len 50
  -min_qual_mean 25 -ns_max_p 1 -noniupac -lc_method entropy -lc_threshold 70
  -out_format 3 -out_good good_reads.fastq -out_bad bad_reads.fastq
```

This left 261,459,213 of the overall 307,223,097 reads (85%). The more reads there are available, the more AS events can be detected and the higher is the "raw" AS rate of a species, i.e. dividing all detected AS events by the number of genes. On the other hand, random sampling normalizes the rate. This is why we found a slightly negative correlation (-0.31) of the number of reads with the ratio between the AS rate from random sampling and the raw AS rate. For the species with the most EST data in our study (*A. benhamiae*, 1,040,774 NGS reads), this ratio is 0.3, that means, the AS rate from random sampling (8.2%) is around one third of the raw AS rate (27.4%). Similar, we assume the raw AS rate of *S. pombe* from the Rhind et al. study (8.4% = 433 AS events/5144 genes) is a strong over-estimation of the real rate. We suppose that the rate from random sampling would be much smaller than 2.5% (0.3 x 8.4%). This is because the use of over 250 times more reads than for *A. benhamiae* likely has revealed many AS events with a strong expression bias towards one isoform, which are not accounted for in random sampling. Thus, the AS rate of *S. pombe* is clearly lower the mean AS rate of non-yeast Ascomycota (7.2%).

[3]For explanation of PRINSEQ parameters see http://prinseq.sourceforge.net/manual.html

Supplementary Figures

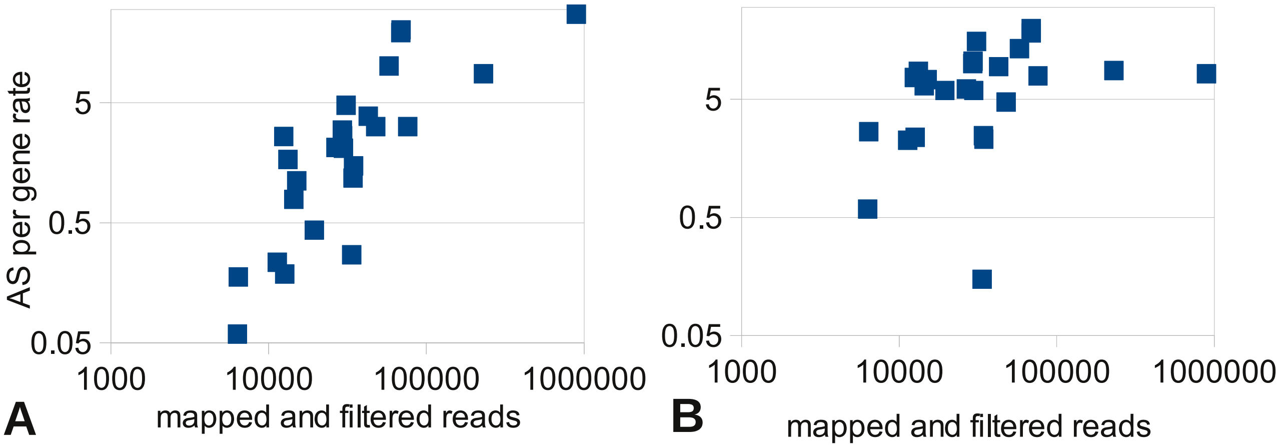

Figure S1: **Dependence of alternative splicing rates on read amounts.** Each point in the diagrams represents data of one species. (A) AS rate from dividing the number of AS events by the annotated gene number. (B) AS rate as average from repeated random sampling of transcripts. Note the logarithmic scaling of the axes.

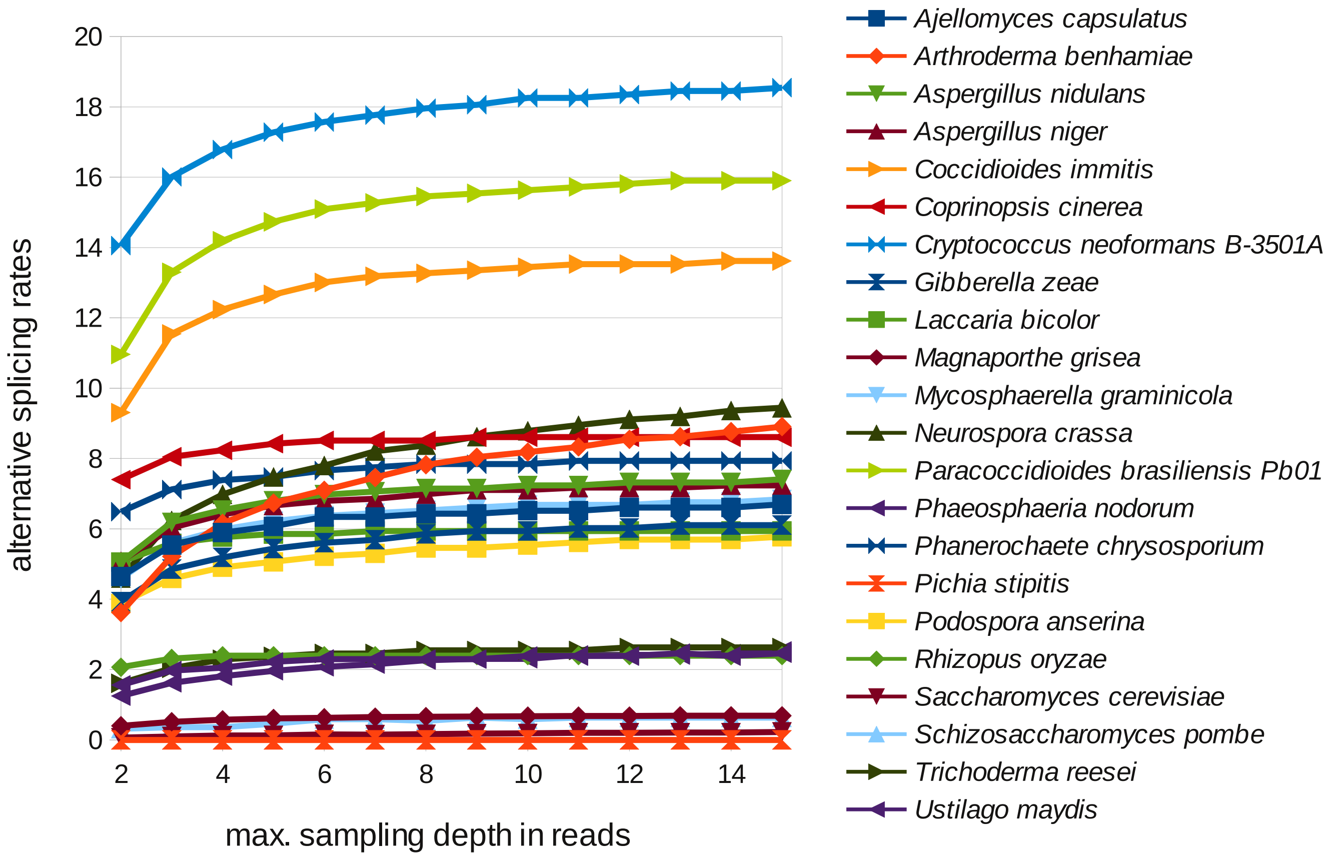

Figure S2: **AS rates versus maximal randomly sampled reads per locus.**

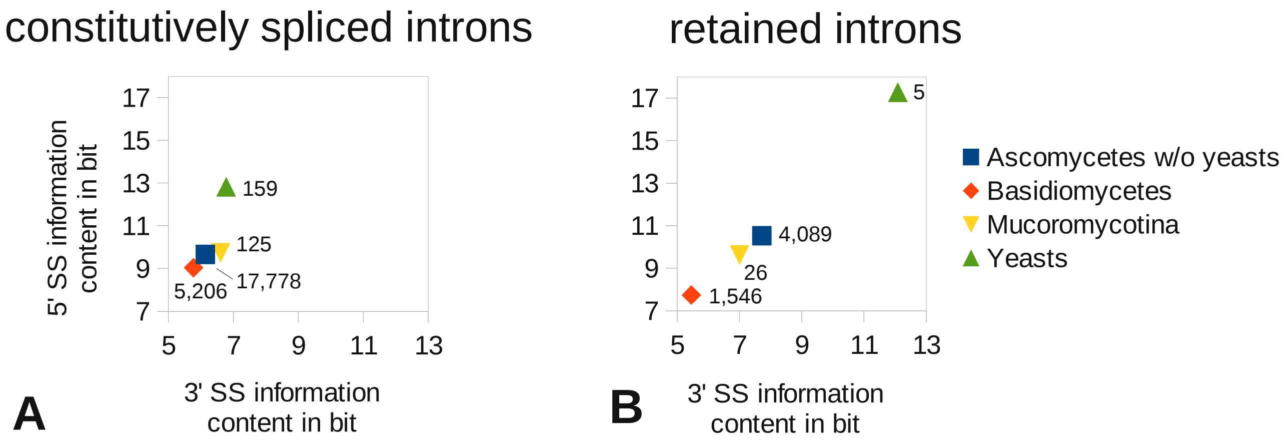

Figure S3: **Mean splice site conservation** Splice site (SS) conservation is calculated as information content in bits as follows: For each intron and SS type (3' and 5'), the SS regions of all introns were stacked. The logarithm of each base's frequency times the frequency is added and summed over all sequence positions. Numbers of underlying introns are noted besides the chart symbols.

References

[1] Aidé MA (2009) Chapter 4–histoplasmosis. J Bras Pneumol 35: 1145–1151.

[2] Burmester A, Shelest E, Gloeckner G, Heddergott C, Schindler S, et al. (2011) Comparative and functional genomics provide insights into the pathogenicity of dermatophytic fungi. Genome Biol 12: R7.

[3] Bennett JW (2009) Aspergillus: a primer for the novice. Med Mycol 47 Suppl 1: S5–12.

[4] Choquer M, Fournier E, Kunz C, Levis C, Pradier JM, et al. (2007) Botrytis cinerea virulence factors: new insights into a necrotrophic and polyphageous pathogen. FEMS Microbiol Lett 277: 1–10.

[5] de Deus Filho A (2009) Chapter 2: coccidioidomycosis. J Bras Pneumol 35: 920–930.

[6] Michielse CB, Rep M (2009) Pathogen profile update: Fusarium oxysporum. Mol Plant Pathol 10: 311–324.

[7] McMullen M, Jones R, Gallenberg D (1997) Scab of wheat and barley: A re-emerging disease of devastating impact. Plant Disease 81: 1340 - 1348.

[8] Ribot C, Hirsch J, Balzergue S, Tharreau D, Nottéghem JL, et al. (2008) Susceptibility of rice to the blast fungus, magnaporthe grisea. J Plant Physiol 165: 114–124.

[9] Bowler J, Scott E, Tailor R, Scalliet G, Ray J, et al. (2010) New capabilities for mycosphaerella graminicola research. Mol Plant Pathol 11: 691–704.

[10] Ray CG, Ryan KJ, editors (2004) Sherris Medical Microbiology. MCGRAW-HILL, MEDICAL PUBLISHING DIVISION, 4 edition.

[11] Stukenbrock EH, Banke S, McDonald BA (2006) Global migration patterns in the fungal wheat pathogen phaeosphaeria nodorum. Mol Ecol 15: 2895–2904.

[12] Agbogbo FK, Coward-Kelly G (2008) Cellulosic ethanol production using the naturally occurring xylose-fermenting yeast, pichia stipitis. Biotechnol Lett 30: 1515–1524.

[13] Paoletti M, Saupe SJ (2008) The genome sequence of podospora anserina, a classic model fungus. Genome Biol 9: 223.

[14] Olsson I, Bjerling P (2011) Advancing our understanding of functional genome organisation through studies in the fission yeast. Curr Genet 57: 1–12.

[15] Hegedus DD, Rimmer SR (2005) Sclerotinia sclerotiorum: when "to be or not to be" a pathogen? FEMS Microbiol Lett 251: 177–184.

[16] Schuster A, Schmoll M (2010) Biology and biotechnology of trichoderma. Appl Microbiol Biotechnol 87: 787–799.

[17] McKnight KH, McKnight VB, Peterson RT (1998) A Field Guide to Mushrooms: North America. Houghton Mifflin Harcourt.

[18] Sidrim JJC, Costa AKF, Cordeiro RA, Brilhante RSN, Moura FEA, et al. (2010) Molecular methods for the diagnosis and characterization of cryptococcus: a review. Can J Microbiol 56: 445–458.

[19] Martin F, Nehls U (2009) Harnessing ectomycorrhizal genomics for ecological insights. Curr Opin Plant Biol 12: 508–515.

[20] Singh D, Chen S (2008) The white-rot fungus phanerochaete chrysosporium: conditions for the production of lignin-degrading enzymes. Appl Microbiol Biotechnol 81: 399–417.

[21] Brefort T, Doehlemann G, Mendoza-Mendoza A, Reissmann S, Djamei A, et al. (2009) Ustilago maydis as a pathogen. Annu Rev Phytopathol 47: 423–445.

[22] Gonzalez CE, Rinaldi MG, Sugar AM (2002) Zygomycosis. Infect Dis Clin North Am 16: 895–914, vi.

[23] Coenen TM, Aughton P, Verhagen H (1997) Safety evaluation of lipase derived from rhizopus oryzae: summary of toxicological data. Food Chem Toxicol 35: 315–322.

[24] Rhind N, Chen Z, Yassour M, Thompson DA, Haas BJ, et al. (2011) Comparative functional genomics of the fission yeasts. Science 332: 930–936.

[25] NCBI (2009). SRA Handbook [Internet]. URL `http://www.ncbi.nlm.nih.gov/books/NBK47540/`.

[26] Schmieder R, Edwards R (2011) Quality control and preprocessing of metagenomic datasets. Bioinformatics 27: 863–864.
